# The dark side of sexism in Argentina: Psychometric properties of the Short Dark Triad Personality measure and its relation with ambivalent sexism

**DOI:** 10.3389/fpsyg.2022.962934

**Published:** 2022-11-07

**Authors:** Paula Bria, Edgardo Etchezahar, Joaquín Ungaretti, Talía Gómez Yepes

**Affiliations:** ^1^Faculty of Psychology, University of Buenos Aires, Buenos Aires, Argentina; ^2^Faculty of Education, Valencian International University, Castelló de la Plana, Spain

**Keywords:** Dark Triad Personality, Ambivalent Sexism, Prejudice, Narcissism, Psychopathy, Machiavellianism

## Abstract

One of the main evaluation instruments of the dark side of personality has been the Short Dark Triad of Personality (SD3), that includes Machiavellianism, Narcissism and Psychopathy traits. Although other adaptations of this scale have been made in several countries, its psychometric properties have never been tested in Argentina. Different studies addressed that dark triad scores are related to different expressions of sexist prejudice. One of the issues that have been traditionally considered to understand intimate, yet unequal relationships between men and women, is prejudice toward women. Ambivalent sexism combines two types of sexist attitudes: hostile, and benevolent sexism. While hostile sexism involves attitudes of outright intolerance towards women, benevolent sexism is defined as a set of attitudes that comprises the perception of women in a positive emotional tone. The aim of the study was to analyze the Dark Triad of Personality scale in the Argentinian context and its relationships whit ambivalent sexism. A total of 1,198 individuals residing in different regions of Argentina participated, from different genders (woman = 59.5%), from 18 to 75 years old (*M* = 45.17 *SD* = 15.08). Main results indicated adequate psychometric properties for the Short Dark Triad of Personality scale in the Argentinian context. In addition, the three traits of the dark triad were significantly related to hostile and benevolent sexism, which indicate that one of the variables to keep addressing in order to comprise and eventually reduce prejudice towards women would be the dark triad of personality. Main results are discussed, in order to strengthen the understanding of the relationships between the dark triad and ambivalent sexism.

## Introduction

Gender inequalities are an existing phenomenon in general (Camou et al., 2017), and Argentina is not an exception in that topic with official data indicating that there were 251 victims of femicide in 2020, and that in 84% of cases the victim knew the perpetrator (National Registry of Femicides of Argentinian Justice, 2020). Sexism is one of the prejudices that presents the highest levels in its hostile and benevolent forms in Argentina: it has been observed to a greater degree in males, and seven out of 10 people state that they have been victims of sexual harassment or know someone who was, and female and younger people report this to a greater extent (Etchezahar et al., 2020). The possible relationship of the dark personality with prejudice towards women and its different manifestations has been relevant in the scientific literature, since numerous research have shown the relationship between dimensions of the dark triad and problems of gender inequality, which they are more than manifestations of prejudiced attitudes: for example, gender-based violence (Holtzworth-Munroe et al., 2003) and acts of sexual aggression (Hersh and Gray-Little, 1998).

Different theoretical and methodological frames converge on personality psychology (Romero Triñanes, 2002). However, this discipline has a dense and controversial history (Ibáñez and Galdón, 1985; Pelechano, 1993; McAdams, 1997; Winter and Barenbaum, 1999; Dumont, 2010). Although the history of personality psychology has its beginning in the early twentieth century (Allport, 1937), dark-but normal-traits have begun to arouse interest recently (Nohales Nieto, 2015).

One of the main goals of personality psychologists interested in the dark side of personality has been to develop a series of measurable constructs to assess individual differences in traits (Hare, 2003). One of the most interesting attempts is the Dark Triad of Personality, initially developed and defined by Paulhus and Williams (2002) as three overlapping personality traits related to harming or exploiting others. This triad is composed of *Machiavellianism-manipulation*, self-interest, cynicism, opportunism and exploitation of others-, *Narcissism-egoism*, need to be admired, lack of empathy, self-admiration, and grandiosity-and *Psychopathy-impulsivity*, callousness, antisocial behaviors, lack of remorse or guilt–(Jonason et al., 2012b). Psychopathy is often considered the “darkest” of the dark triad traits: it involves meanness, disinhibition, and boldness (Patrick et al., 2009). In this order, psychopaths have more disregard for others, operationalized for example in behaviors such as sadism (Carton and Egan, 2017), or bullying (Baughman et al., 2012). On the other hand, narcissism is studied also as a regular personality trait (Miller and Campbell, 2008; Morf and Rhodewalt, 2001), defining persons with an extremely positive image of themselves, yet vulnerable at the same time. Machiavellianism has been defined as a trait that consists of manipulating and using others as tools to achieve one’s own goals, making interpersonal strategies that are related to self-interest and deceiving others (Paulhus and Williams, 2002). As a result of pioneering studies, Machiavellianism was found to include a cynical worldview, lack of morality, and manipulation (Fehr et al., 1992). Following the claims of Sun Tzu-a first century military strategist- (Tzu, 1998) a more recent review has added planning, coalition building, and reputation building to Machiavellianism (Jones and Paulhus, 2009). These latter characteristics are critical in distinguishing psychopathy from Machiavellianism. While psychopaths are impulsive, abandon their bonds and do not find their reputation relevant (Hare and Neumann, 2008), Machiavellians are forward-looking, plan, build and try to maintain a positive reputation. That is, Machiavellians are strategic rather than impulsive (Jones and Paulhus, 2011a), and they avoid manipulating people in their family (Barber, 1998) as well as engaging in any other behavior that might undermine their reputation (Shepperd and Socherman, 1997).

Similar to narcissism, psychopathic individuals are characterized by traits such as callousness and lack of empathy, but psychopathy differs from narcissism in its relations to disinhibition, as disinhibition is in almost all conceptions of psychopathy (Cleckley, 1941; Hare, 1980). Psychopaths manifest their callousness in the short term (Visser et al., 2010; Jones and Paulhus, 2011a). For example, they lie for immediate rewards, even if those lies compromise their long-term interests (Jones and Paulhus, 2010). However, the impulsivity element of psychopathy is the key to distinguishing it from Machiavellianism (Newman et al., 2005; Hicks et al., 2007). In this sense, people who possess high dark characteristics have a dysfunction in interpersonal relations due to the insufficient concern for others. In addition, it should be pointed out that the dark triad traits are not considered as clinical traits, and they are not related to clinical disorders (Lyons, 2019). Since some of the above traits overlap, it is not surprising that the three of them correlate with each other, creating an insensitive and manipulative personal style (Jones and Paulhus, 2010).

Therefore, these three traits imply a lack of respect for societal norms, that frequently conducts to social indiscrete behaviors as thieving, lying, tricking people, or manipulating.

### The evaluation of the Short Dark Triad and gender differences

In order to systematically gather evidence on these personality traits, the *Short Dark Triad (SD3)* scale was developed by Jones and Paulhus in 2014 due to a lack of valid and reliable instruments to measure the dark triad of personality in an agile way. Before creating the SD3, the authors carried out four studies that examined the structure, validity, and reliability of the SD3 scale in general population samples recruited through online surveys. The result was an initial 27-item and three factors scale (Jones and Paulhus, 2011b) that correlated positively with the scales traditionally used to measure psychopathy (*SRP-III*; Williams et al., 2007; Paulhus et al., 2015), narcissism (*NPI*; Raskin and Hall, 1981), and Machiavellianism (*Mach-IV*; Christie and Geis, 1970). Finally, the subscales of the SD3 showed good psychometric properties in all the subsamples tested.

In a recent study Pineda et al. (2020) adapted the scale in Spain with adequate psychometric properties and significant differences were found in the narcissism scores according to participants gender where male participants had higher scores than females. Furthermore, statistically significant differences were found according to gender for psychopathy and Machiavellianism. However, other adaptations of the SD3 have been made in countries such as Germany (Malesza et al., 2019), Serbia (Dinić et al., 2018), France (Gamache et al., 2018), Turkey (Özsoy et al., 2017), Italy (Somma et al., 2019), China (Zhang et al., 2020), and Colombia (Nohales Nieto, 2015). In the German version (Malesza et al., 2019), a three-factor structure has been found, which is consistent with the original version (Paulhus and Williams, 2002; Jones and Paulhus, 2014). This structure had an adequate fit while the one-factor model did not show an acceptable fit. Consistently with literature on the topic (Furnham et al., 2013), men scored higher than women on narcissism, and psychopathy, as well as in the complete scale. Similar results were found in the Serbian version (Dinić et al., 2018): in two studies men scored higher in Machiavellianism, psychopathy, and narcissism. Likewise, the fit of the three-factor model was more adequate than the one-factor. In Turkey, results also showed that the three-factor model fits adequately and men score higher than women in psychopathy and Machiavellianism, but there was not a statistically significant difference in narcissism (Dinić et al., 2018).

### Dark Triad and sexism

To date, previous research has associated Machiavellianism with hostile sexual attitudes, with promiscuity, and sexual deceptions, such as the disclosure of sexual secrets, feigned love, induced intoxication to obtain sex, and endorsement of the use of force for sexual purposes (McHoskey, 2001; Jonason et al., 2009). Nevertheless, results indicate that psychopathy is related to breakups and relationship difficulties (Han et al., 2003; Savard et al., 2006), short-term or casual sexual relationships (Jonason et al., 2012a), and infidelities (Egan and Angus, 2004). Furthermore, both sexism and Machiavellianism have been found to be higher at the same time, and this could indicate that they share a common factor (Pina et al., 2017). On the other hand, antecedents indicate that narcissistic individuals, whether male or female, tend to endorse a wide range of biases (Hodson et al., 2009), including sexism (Keiller, 2010). Recent studies addressed the prevention and reduction of sexism in men (Good and Woodzicka, 2009; Becker et al., 2014; Kilmartin et al., 2015) and affirm that sexism is nothing more than a set of learned attitudes. According to Gluck et al. (2020), if the traits of the dark triad are homologous to learned sexism, it can be reduced or prevented. One robust finding to emerge from the limited existing literature is that males scored higher than females in both dark personality traits and also in ambivalent sexism, and that hostile sexism mediated the relationship between gender and the dark triad score. In addition to this, results showed that sexist prejudice predicts dark triad scores, suggesting that sexism could be one of the sources of dark personality traits (Gluck et al., 2020). Although, as seen, there are some studies that relate psychopathy, narcissism and Machiavellianism to sexism, or to phenomena that are a product of this cognitive bias, there is still a considerable gap in the study of ambivalent sexism and dark personality. And even more so in the Argentinian context. For this reason, it is relevant to adapt the short scale that measures the dark personality triad, in order to initiate the analysis of the relationship between these two psychological constructs in Argentina. This is essential in order to have another tool that allows not only to measure this peculiar type of normal personality, but also its use in favor of social change.

### The present study

Also, previous research indicate that gender violence is related to narcissistic (Echeburúa et al., 2009; Esbec and Echeburúa, 2010; Torres et al., 2013) and antisocial syndromes usually associated with psychopathy (Echeburúa et al., 2009; Esbec and Echeburúa, 2010). The lack of empathy inherent to narcissism predicts ambivalent sexism (Hellmer et al., 2018) and Machiavellianism are strong and positively related to the sexist prejudice (Navas et al., 2020). In addition, dark personality was related to intimate partner violence (Pineda et al., 2021), and narcissism to positive attitudes towards courtship violence (Erdem and Sahin, 2017). The first hypothesis of the study was that the three dimensions of the dark personality are significantly related and it is possible to evaluate them through the Short Dark Triad Scale. The second hypothesis was that the three dark triad traits are significantly related to both dimensions of ambivalent sexism (hostile sexism and benevolent sexism).

## Materials and methods

### Participants

A cross-sectional study was conducted and a geolocated online survey was administered, with a quota sampling (Lliyasu and Etikan, 2021), according to three-quota proportional fixation: gender, age and Argentina’s geographic regions (see Table 1). The sample size was calculated for a 95% CI, with a tolerated margin error of 5% based on a population National Survey (INDEC, 2020). Overall, 1,198 adults from different regions of Argentina participated in the study, aged between 18 and 75 years old (*M* = 45.17; *SD* = 15.08); 59.5% (*n* = 713) were female, 7.3% (*n* = 87) finished primary school, 22% (*n* = 264) high school, 42.7% (*n* = 512) *terciario* education [comparable to an Associate’s Degree], and 28% (*n* = 335) university education. No cases were dismissed from the total sample due to missing values, according to Tabachnick et al. (2007) criteria (cut off >5%).

**Table 1:** Distribution of participants by geographic region.

Geographic region	ƒ	%
Buenos Aires City	140	11.7
Buenos Aires province	606	50.6
Region 1 (Córdoba, Santa Fe, Mendoza, San Luis, San Juan)	259	21.6
Region 2 (Salta, Jujuy, Catamarca, La Rioja, Santiago del Estero, Tucumán)	65	5.4
Region 3 (Misiones, Chaco, Formosa, Entre Ríos, Corrientes)	69	5.8
Patagonia Region (La Pampa, Río Negro, Neuquén, Chubut, Santa Cruz, Tierra del Fuego)	59	4.9
Total	1,198	100

### Instruments

The survey included the following material. *Short scale of the Dark Triad of Personality* (SD3; Jones and Paulhus, 2014): the original version of the scale was translated to Spanish. Translations were performed in accordance with the guidelines for cross-cultural research as recommended by the International Test Commission (Hambleton, 2001): First, the questionnaire was forward-translated from the source language (English) into Spanish by bilingual native speakers with previous experience in psychological test translations. Second, an independent bilingual person back-translated the resulting version into English. Third, after a discussing the differences between the original and the back-translated questionnaire, the final version of the SD3 adapted to Spanish was reached. It is made up of 27 items, with a Likert-type response format with five anchors, from 1 (*totally disagree*) to 5 (*totally agree*). There are three dimensions, and each one has nine items: Machiavellianism (e.g., “Most people can be manipulated”), Narcissism (e.g., *“*I know that I am special because everyone keeps telling me so”), and Psychopathy (e.g., “Payback needs to be quick and nasty”).

*Ambivalent sexism scale (ASI):* the Argentinian adaptation (Etchezahar, 2012) of the *Ambivalent Sexism Inventory* (ASI; Glick and Fiske, 1996) was used, which has proven to be an adequate instrument for the evaluation of sexism in different sociocultural contexts (Rudman and Glick, 2012). The inventory has a Likert-type response format with five options, on a continuum that goes from 1 = *Strongly disagree* to 5 = *Strongly agree.* It includes items that refer to Hostile Sexism (e.g., “Women are very easily offended”) and Benevolent Sexism (e.g., “A man is incomplete without a woman”). The reliability levels in the present study were adequate for the total scale, as well as for the dimensions that compose it: benevolent sexism (*α* = 0.90) and hostile sexism (*α* = 0.92).

*Questionnaire of sociodemographic variables* (*ad-hoc*): participants were asked about gender, age, province of residence and educational level.

### Procedure

Participants received no compensation for participation and their consent was requested. They were also informed that data derived from this research will be used exclusively for academic-scientific purposes, under Argentinian National Law 25.326 on the protection of personal data and anonymity insurance. The survey could be fully answered in 15 min. The geolocated survey service has been configured to guarantee that the same IP cannot send two responses to the form. Likewise, an email address was offered for any questions regarding the research.

### Data analysis

To carry out the analyses, SPSS *software-version* 21-and AMOS program were used. The descriptive statistics of the items-mean, standard deviation, kurtosis, asymmetry-were calculated and analyzed. The factor structure of the short scale of the dark triad of personality was verified through a confirmatory factor analysis. In addition, within each subscale-Machiavellianism, narcissism and psychopathy-, item discrimination was analyzed using the item-dimension correlation, and *Cronbach’s Alpha* if item is deleted. To test the internal consistency of each scale, Cronbach’s Alpha statistic was used. Then, a confirmatory factor analysis was carried out to find out if the responses of the people who participated were adjusted to the three-dimensional model that theoretically possesses the construct of the dark triad of personality. Likewise, for the analysis of the external validity of the scale, correlations were made between the dark triad of personality and ambivalent sexism using *Pearson’s r statistic.* Finally, a *t-test was performed* for independent samples, in order to verify if there were differences in the levels of the dark triad of personality according to gender.

## Results

A total of 27 items from the original SD3 scale (Paulhus and Williams, 2002) was tested to analyze the first hypothesis of the study. According to the validity and reliability criteria presented below, the 15 items that make up the final SD3 scale were determined. Table 2 shows the final wording of each of the items and the descriptive statistics (mean, standard deviation, asymmetry, kurtosis, item-total correlation and Cronbach’s alpha if the item is deleted).

**Table 2:** Descriptive statistics of the items of the Short Scale of the Dark Triad.

*items*	*M*	*SD*	*S*	*K*	rjx	*α*.-*x*
*Machiavellianism* (*α* = 0.73)						
M1. I like to use clever manipulation to get my way. (Me gusta usar la manipulación de manera inteligente para salirme con la mía.)	1.92	1.27	1.06	−0.27	0.490	0.692
M2. Whatever it takes, you must get the important people on your side. (Cueste lo que cueste, debés conseguir que la gente importante esté de tu lado.)	1.85	1.15	1.07	−0.03	0.525	0.681
M3. It’s wise to keep track of information that you can use against people later. (Es prudente mantener un registro de la información que podés usar contra la gente en el futuro.)	1.99	1.32	0.99	−0.39	0.534	0.675
M4. You should wait for the right time to get back at people. (Deberías esperar el momento adecuado para vengarte de la gente.)	1.80	1.27	1.28	0.24	0.523	0.680
M5. Most people can be manipulated. (La mayoría de la gente puede ser manipulada.)	3.47	1.34	−0.66	−0.78	0.418	0.720
*Narcissism* (*α* = 0.72)						
N1. People see me as a natural leader. (La gente me ve como un líder natural.)	2.67	1.29	−0.04	−1.24	0.474	0.681
N2. I like being the center of attention. (Me gusta ser el centro de atención.)	1.85	1.16	1.07	−0.10	0.546	0.655
N3. I know that I am special because everyone keeps telling me so. (Sé que soy especial porque todo el mundo me lo dice.)	2.09	1.21	0.63	−0.85	0.467	0.684
N4. I like to get compliments. (Me gusta que me hagan cumplidos.)	2.88	1.35	−0.15	−1.27	0.366	0.726
N5. I am a person who stands out above the average. (Soy una persona que se destaca sobre el promedio.)	2.67	1.28	−0.04	−1.22	0.583	0.636
*Psychopathy* (*α* = 0.71)						
P1. I like to get revenge on authorities. (Me gusta vengarme de las autoridades.)	1.82	1.20	1.19	0.14	0.477	0.652
P2. Payback needs to be quick and nasty. (La venganza tiene que ser rápida y desagradable.)	1.58	1.02	1.63	1.73	0.473	0.657
P3. It’s true that I can be mean to others. (Es cierto que puedo ser malo/a con los demás.)	2.45	1.39	0.30	−1.37	0.453	0.669
P4. People who mess with me always regret it. (La gente que se mete conmigo siempre se arrepiente.)	1.85	1.18	1.08	−0.08	0.552	0.620
P5. I’ll say anything to get what I want. (Diré lo que sea para conseguir lo que quiero.)	1.50	0.98	2.00	3.07	0.383	0.688

*M*, mean; *SD*, Standard deviation; *S*, Asymmetry; *K*, Kurtosis; rjx, Item-total correlation; *α*.-x, Alpha if element is deleted.

In general, all the items contribute adequately to the set of each subscale, since they present an adequate correlation with the total scale, and the reliability of each subscale it is not improved by eliminating any element. According to (a) the levels of kurtosis and asymmetry of each item (−1.5 < *x* < 1.5) (five items were eliminated due to excess of kurtosis), (b) the analysis of item-total correlation of each dimension-narcissism, psychopathy, Machiavellianism–(> 0.40) (five items were removed due to low correlation with their own factor), and (c) Cronbach’s alpha if item deleted-the elimination of any item increases the internal consistency of the scale-(two items were deleted because they decreased the internal consistency of the dimension), a scale of 15 items was obtained, with five items for each dimension of the dark triad of personality. The internal consistency of Machiavellianism (*α* = 0.73), narcissism (*α* = 0.72), and psychopathy (*α* = 0.71) dimensions were adequate.

**Figure 1:**
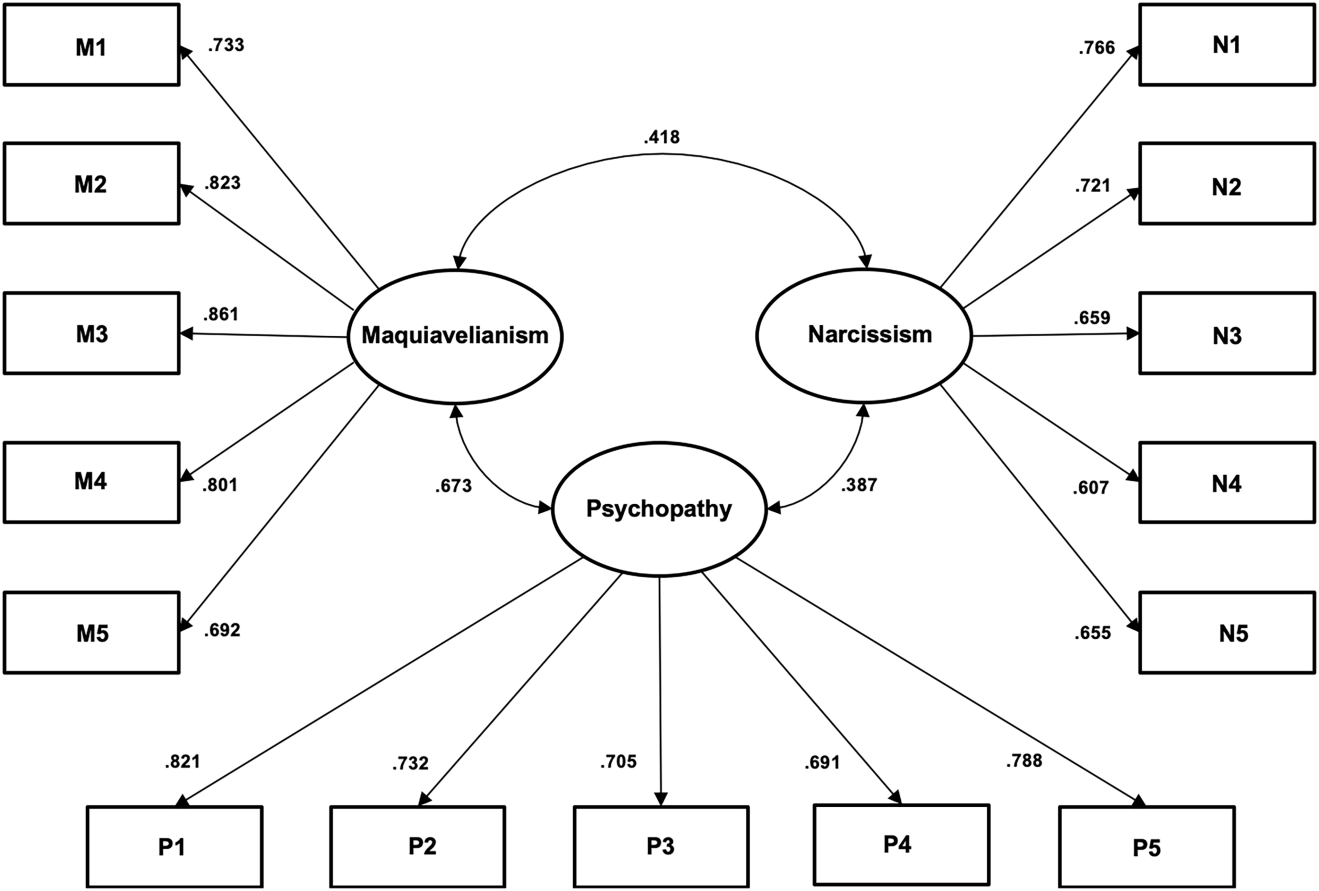
Short Dark Triad Validation Structural Equation Model.

Then, it was analyzed if the data fits the three-dimensional model properly, as seen in Figure 1. A confirmatory factorial analysis was carried out, which showed adequate construct validity (AGFI = 0.92; RMSEA = 0.069). Machiavellianism subscale includes five items of the seven considered in the original scale by Jones and Paulhus (2014). Regarding narcissism, five items are included: two of the seven original items, while the remaining three correspond to inverted versions of the original items. Finally, the psychopathy subscale retains five of the original seven items.

Subsequently, the second hypothesis of the study was tested by addressing the relation between the dark triad of personality-Narcissism, Machiavellianism, and Psychopathy-and ambivalent sexism-hostile sexism and benevolent sexism-. Pearson’s correlation coefficient was used and, as seen in Table 3, positive relationships between Machiavellianism, Narcissism, Psychopathy and the two dimensions of ambivalent sexism were found. Machiavellianism was the only one that obtained a higher moderate correlation with both hostile (*r* = 0.306) and benevolent sexism (*r* = 0.296).

**Table 3:** Relationships between the dark triad of personality and ambivalent sexism.

	*α*	1	2	3	4	5
1. Machiavellianism	0.73	–	0.418^**^	0.673^**^	0.306^**^	0.296^**^
2. Narcissism	0.72		–	0.387^**^	0.195^**^	0.177^**^
3. Psychopathy	0.71			–	0.274^**^	0.220^**^
4. Hostile Sexism	0.92				–	0.572^**^
5. Benevolent Sexism	0.90					–

** *p* < 0.01; *α* = Cronbach’s Alpha.

On the other hand, statistically significant differences between men and women in terms of levels of Machiavellianism (*t* = −3.562; *p* < 0.01) and Psychopathy (*t* = −4.453; *p* < 0.01) were identified. Likewise, males obtained higher scores in Machiavellianism (*M* = 2.06; *SD* = 0.94) than females (*M* = 1.83; *SD = 0*.81). Similarly, those who considered themselves masculine (*M* = 1.99; *SD = 0*.83) obtained higher scores in Psychopathy than those who considered themselves feminine (*M* = 1.74; *SD* = 0.73). However, no differences were found regarding Narcissism.

## Discussion

Based on the results obtained, it is possible to affirm that the adaptation of the short scale of the dark triad of personality presents adequate psychometric properties in the Argentinian context, with evidences of validity and reliability. Five of the original items of the Machiavellianism scale were suitable for the present adaptation, while the narcissism subscale remained with five items. Likewise, the most appropriate items corresponding to psychopathy were five of nine that include the original version. The remaining items that were analyzed were rejected because they presented a low item-dimension correlation. Once items were established, scale dimensionality was analyzed in order to find out if the model with the best fit to the data collected was three-dimensional as originally proposed by Jones and Paulhus (2014), or if the model fitted better unidimensionally, as observed in results from other countries (e.g., Dinić et al., 2018; Gamache et al., 2018; Malesza et al., 2019; Pineda et al., 2020). Better fit indexes were found for the three correlated dimensions model-Narcissism, Machiavellianism, and Psychopathy. Cronbach’s alpha turned out to be adequate for the three dimensions, corroborating the first hypothesis of our study. These results are similar to those using the SD3 scale in other contexts, including Spanish-speaking ones (e.g., Nohales Nieto, 2015; Pineda et al., 2020). It can therefore be concluded that the assumption of each trait of the dark triad framework as unique is legitimate and superior over mapping it as a single construct.

This is further supported by our finding that the genders scored differentially on these traits, with self-perceived male participants having higher scores in most SD3 dimensions, except for Narcissism, as was shown in the study by Dinić et al. (2018). More specifically, it was cross-culturally shown (Furnham et al., 2013) that males scored significantly higher on Machiavellianism and Psychopathy than females, while no gender wise differences were observed for narcissism. If all traits would measure the same underlying construct, however, it might be expected that males have higher scores across all SD3 traits.

Regarding the second hypothesis, the relationships between the dark triad of personality and ambivalent sexism, statistically significant relationships between both constructs and their corresponding subscales were found. These findings are in line with the scientific literature that indicate, for example, that hostile sexism is associated with Machiavellianism (McHoskey, 2001; Jonason et al., 2009), and that the greater the sexist prejudice, the greater the scores in dark triad (Gluck et al., 2020).

## Conclusion

Although there are studies that relate Psychopathy, Narcissism, and Machiavellianism to sexism, there is still a gap in the study of ambivalent sexism and dark personality in the Argentinian context. For this reason, it was relevant to adapt the SD3 scale, in order to increase the analysis of the relationship between these two psychological constructs in the Argentinian context. Sexism is a very serious problem in Argentina and over the world. It is important to know which ones are potential variables related to this phenomenon–such as dark triad as previous literature suggests-and assess them. Furthermore, it is a major issue to have the proper adaptations of the instruments that operationalize the constructs related to sexism according to the antecedents. In addition to that, adapted instruments are valuable tools to evaluate the results of interventions, for example, to reduce sexism.

In this framework, the data of the present study has been collected in all regions in Argentina, that allowed the assessment of the SD3 validity in Argentina based on Jones and Paulhus (2014) original scale. Then, this research determined that the dark personality traits were related to ambivalent sexism. Finally, dark triad personality scores were compared-both globally and by subscale–according to gender, identifying statistically significant differences in Machiavellianism and Psychopathy.

### Limitations

The present study has different limitations. First, all the measures were completed through self-report questionaries in a non-experimental design. While we do not see this as problematic, it would be important to use other kind of measures and research designs (e.g., behavioral measures, experimental designs) in order to provide stronger evidence for the validity of these constructs. Second, participants were recruited from social media and directed to online survey platforms. While there is previous evidence supporting these kind of procedures (Goodman et al., 2013; Peer et al., 2017), maybe more ecologically samples would increase the validity of the results in our study. Third, convenience samples like the ones in this study, requires further research to assess the generalizability of the findings to a wider range of people in Argentina. Finally, it is considered essential on this topic to evaluate the short scale of the dark triad of personality considering not only the geographical distribution of the sample, but also age and socioeconomic status, among others.

### Future directions

Furthermore, future research should analyze the relationship between dark personality traits and other variables associated with sexism, such as gender role ideology (Moya et al., 2006) or partner ideals (Fletcher and Simpson, 2000). However, future research should also address the serious problem of sexism conducting psychosocial interventions to reduce prejudice against women, taking into account the adaptations of the instruments involved in the assessment of the results of the intervention but also in the diagnosis of the problem. Finally, as noted above, further research on this topic might benefit from a greater focus on behavioral outcomes, demonstrating that these measures predict differences in behavior between individuals.

## Data availability statement

The raw data supporting the conclusions of this article will be made available by the authors, without undue reservation.

## Ethics statement

Ethical review and approval was not required for the study on human participants in accordance with the local legislation and institutional requirements. Written informed consent for participation was not required for this study in accordance with the national legislation and the institutional requirements.

## Author contributions

PB and EE contributed to design the present study. JU and EE organized the database. PB, EE, and JU did the statistical analysis. PB and TG wrote the first draft of the article. PB, EE, JU, and TG wrote the different sections. All authors contributed to the article and approved the submitted version.

## Conflict of interest

The authors declare that the research was conducted in the absence of any commercial or financial relationships that could be construed as a potential conflict of interest.

## Publisher’s note

All claims expressed in this article are solely those of the authors and do not necessarily represent those of their affiliated organizations, or those of the publisher, the editors and the reviewers. Any product that may be evaluated in this article, or claim that may be made by its manufacturer, is not guaranteed or endorsed by the publisher.
